# LncRNA-Gm9795 promotes inflammation in non-alcoholic steatohepatitis via NF-$\kappa {}$B/JNK pathway by endoplasmic reticulum stress

**DOI:** 10.1186/s12967-021-02769-7

**Published:** 2021-03-09

**Authors:** Liangying Ye, Dan Zhao, Yangzhi Xu, Jiaen Lin, Jiahui Xu, Kunyuan Wang, Zhanhui Ye, Yufeng Luo, Shiming Liu, Hui Yang

**Affiliations:** 1 Department of Gastroenterology, The Second Affiliated Hospital of Guangzhou Medical University, Guangzhou, China; 2 Guangzhou Institute of Cardiovascular Disease, The Second Affiliated Hospital of Guangzhou Medical University, Guangzhou, China

**Keywords:** NAFLD, NASH, Non coding RNA, Microarrays, ERS

## Abstract

**Background:**

Non-alcoholic steatohepatitis (NASH) is a key stage in leading development of non-alcoholic simple fatty liver (NAFL) into cirrhosis and even liver cancer. This study aimed at exploring the lncRNAs expression profile in NASH and the biological function of a novel LncRNA-gm9795.

**Methods:**

Microarray analysis was performed to compare the expression profiles of lncRNAs in the liver of NASH, NAFLD and normal mice (5 mice for each group). Methionine-choline-deficient Medium (MCD) with Lipopolysaccharide (LPS) or palmitic acid (PA)were used to built NASH cell models. The role and mechanism of LncRNA-gm9795 in NASH were explored by knocking down or over-expressing its expression.

**Results:**

A total of 381 lncRNAs were found to be not only highly expressed in NAFLD, but also is going to go even higher in NASH. A novel LncRNA-gm9795 was significantly highly expressed in liver tissues of NASH animal models and NASH cell models. By staining with Nile red, we found that gm9795 did not affect the fat accumulation of NASH. However, gm9795 in NASH cell models significantly promoted the expression of TNF $\alpha {}$, IL-6, IL-1$\beta {}$, the important inflammatory mediators in NASH. At the same time, we found that gm9795 upregulated the key molecules in endoplasmic reticulum stress (ERS), while NF-$\kappa {}$B/JNK pathways were also activated. When ERS activator Thapsigargin (TG) was introduced in cells with Ggm9757 si-RNA, NF-$\kappa {}$B and JNK pathways were activated. Conversely, ERS inhibitor Tauroursodeoxycholic acid (TUDCA) inhibited NF-kB and JNK pathways in cells with gm9795 overexpression plasmid.

**Conclusion:**

LncRNA-gm9795 promotes inflammatory response in NASH through NF-kB and JNK pathways by ERS, which might provide theoretical basis for revealing the pathogenesis of NASH and discovering new therapeutic targets

## Background

Nowadays, non-alcoholic fatty liver disease (NAFLD) is the most common liver disease in the world, and its estimated prevalence rate is 25% in the general population of Western and Asian countries [1–3]. NAFLD refers to the accumulation of fat in the liver > 5% per liver weight and the occurrence of diffuse hepatocyte bullae steatosis, in the presence of < 10 g of daily alcohol consumption. The pathological process of NAFLD is mainly from non-alcoholic simple fatty liver (NAFL) to non-alcoholic steatohepatitis (NASH), and then to non-alcoholic cirrhosis (NAC) and even to liver cancer [4]. Among them, NASH is an extremely important pathological stage, and blocking the progression of NASH can effectively control the progression of NAFL to cirrhosis and liver cancer. However, the pathogenesis of the inflammatory response in NASH is not fully understood, leading to limited treatment options.

The specific pathogenesis of NAFLD is complex, and it has not yet reached a definite conclusion. One classical hypothesis of the pathogenesis of NAFLD is “two hit theory”.On the basis of the accumulation of lipid, oxidative stress caused by a variety of inflammatory cytokines (such as TNF$\alpha {}$, IL-1β, IL-6, etc.), and the release of fat factors (leptin, adiponectin, etc.), caused liver inflammation and liver cell damage, and eventually progressed to liver fibrosis [5]. Insulin resistance (Insulin to hold, IR) is the core link. However, this theory cannot fully explain the development of lipid deposition in liver cells during steatohepatitis, liver fibrosis and other NAFLD processes.In recent years, the theory of “multi hit” has emphasized the key role of Endoplasmic reticulum stress (ERS) in NAFLD, especially in NASH. Hypoxia, oxidative stress, exogenous substances, and the accumulation of unfolded proteins in the endoplasmic reticulum can all disrupt the endoplasmic reticulum balance, leading to ERS, liver lipid metabolism disorder, hepatocyte inflammatory necrosis and apoptosis, and ultimately the development of NASH [6, 7]. It is not difficult to notice the important role of inflammatory response and endoplasmic reticulum stress in NASH, so inhibiting inflammation and endoplasmic reticulum stress may be a strategy for treating NASH.

Recent studies have shown that long noncoding RNAs (lncRNAs) play a key role in the regulation of gene expression, such as chromatin modification, transcription and post-transcriptional processing, and its abnormal expression is related to many physiological and pathological processes, such as embryonic development, cell proliferation and differentiation, steatosis, oxidative stress, endoplasmic reticulum stress, etc [8–11]. According to the data of multiple studies, there are thousands of differentially expressed lncRNAs in the liver of NAFLD, but only a few of them have been studied. Their role in fat metabolism in NAFLD has been mostly studied, while the role and regulatory mechanism of inflammation in NASH are poorly understood [12]. Therefore, the role of lncRNAs plays in the inflammation of NASH and its specific mechanism are worth studying. In conclusion, it is of great significance to explore the role of lncRNA in the occurrence and development of NSAH for finding reasonable and effective prevention and treatment measures. To explore the lncRNA expression profile in NAFLD and NASH, a microarray analysis was performed to compare the expression profiles of lncRNAs in the liver of NASH, NAFLD and normal mice. And we first found a key long non-coding RNA-gm9795, which is closely related to inflammation in NASH. So far, there have been no studies on LncRNA-gm9795. gm9795 is a pseudogene of StAR-related lipid transfer (START) domain containing 6. It locate at chromosome 10 with a length of 1170 bp. Pseudogene were first introduced by Jacq et al in 1997 when they discovered a copy of the 5S rRNA gene in Xenopus laevis [13]. From then on, a large number of pseudogenes have been gradually found but were considered as non-functional “junk genes” [14]. However, recent studies have revealed that pseudogenes act as key regulators at DNA, RNA or protein level in diverse human disorders, among which pseudogene-derived long non-coding RNA (lncRNA) transcripts are extensively investigated and has been reported to be frequently dysregulated in various types of human cancer [15]. This study aims to explore the role of LncRNA-gm9795 in promoting inflammation in NASH and its molecular mechanism, so as to provide a theoretical basis for revealing the pathogenesis of NASH and discovering new therapeutic targets.

## Materials and methods

### NASH mouse model

Male C57 BLKS/J db/db mice and C57 BLKS/J mice, 6 weeks old, each weighing 18–20 g, were purchased from Changzhou Cavens Laboratory Animal, Co., China. NASH mouse model was established by feeding C57 BLKS/J db/db mice with MCD diet for 4 weeks, NAFL mouse model was constructed by feeding C57 BLKS/J db/db mice with normal diet for 4 weeks, and C57 BLKS/J mice with normal diet as the control group(5 mice for each group) [16]. After four weeks, all mice were sacrificed and their livers were removed. The degree of disease progression was determined by HE staining and IHC with F4/80 of liver pathological sections of mice in each group. NAFLD is characterized by excessive hepatic fat accumulation (more than 5% of hepatocytes), while NASH is accompanied by inflammatory cell infiltration. ALT and AST were also used to assess the degree of liver inflammation.

### RNA preparation and microarray analysis

Total RNA was extracted from the liver of NAFLD, NASH and control mice using Trizol and treated with DNase I (Invitrogen, Carlsbad, CA, United States). The integrity of RNA was assessed by electrophoresis on denatured agarose gel. Mouse LncRNA Microarray V2.0 (Arraystar, Rockville, MD, United States) was used to study the profiles of mouse lncRNAs and mRNA in the liver.

### Cell culture

AML 12 cells were purchased from the Cell Bank of Type Culture Collection (CBTCC, Chinese Academy of Sciences, Shanghai, China) and were cultured in DMEM/F12 (Gibco, Carlsbad, CA) supplemented with 10% fetal bovine serum (FBS, BI, Israel), 1% Insulin-Transferrin-Selenium(ITS, Sciencell, USA), 0.2% Dexamethasone(DM, Sigma-Aldrich, USA) and 1% Penicillin-Streptomycin Solution (PS, Sigma-Aldrich, USA). Cells were maintained at 37 $^\circ {}$C in a humidified 5% CO_2_ atmosphere.

### Primary hepatocytes extraction

After the mice were anesthetized, the mice were fixed to the working platform on the abdomen and the limbs were fixed with tape. The abdomen and chest areas were disinfected and abdominal incisions were made until the liver, portal vein, and inferior vena cava were fully exposed.After closing the superior vena cava, trocars were placed into the inferior vena cava and HBSS was slowly injected. Then the portal vein was cut, and the perfusion velocity was immediately increased to 7–9 ml /min, allowing all HBSS to be perfused through the liver, and the flow rate could increase by 1–2 ml/s. When the HBSS is nearly exhausted, replace it with 70 ml of digestive juice (containing enzymatic digestions) and press the inferior vena cava regularly. When digestion is satisfactory, carefully remove the casing.Separate the liver, immerse the liver in a 10 cm petri dish containing digestive liquid, tear the liver leaves, and the dispersion becomes turbified as the liver tears, discard all remaining solid particles.Blow the suspension three times in the original 10 cm petri dish with a pasteurian tube. The cell suspension was filtered through a 70–75 $\mu {}$m filter and centrifuged in a flattening centrifuge at 50 g for 2 min., the supernatant was absorbed and discarded with sterile pasteurian tube, and then 25 ml cold medium was added to resuspend 50g for centrifugation for 2 min.Repeat washing 2 times.Absorb and discard the supernatant and add 10 mL cold medium, incubate at 37, and change the liquid after 4 h.

### NASH cell model

### MCD model

Spread AML12 cells on the six-hole plates at the density of 1.5$\times {}$10^5^/ hole. After 24 h, the medium in the hole was discarded. 2 ml of DMEM/F12 without FBS was added to each hole for 12 h to starve cells. After 12 h, 2 ml MCD (Gibco, Carlsbad, CA) with 10% FBS medium was added to MCD model group. In the normal control group, 2 ml of DMEM/F12 with 10% FBS, 1% ITS, 0.2% DM and 1% PS was added to each hole. After 12 h, the medium in MCD model group was sucked out of the hole and placed in a sterile 4 ml centrifuge tube. Lipopolysaccharide(LPS) was added to make the working concentration to 1 $\mu {}$g/ml and then refill the hole. The culture was continued for 12 h.

### PA model

AML12 cells were placed in the six-well plates at the density of 1.5$\times {}$10^5^/ well, and the medium was discarded after 24 h. DMEM/F12 without FBS was added to each well for 12 h to starve cells. After that, the control group was cultured with 2 ml of DMEM/F12 with 10%FBS, 1% ITS, 0.2% DM and 1% PS, and the PA model group was stimulated with 2 ml 1 Mm PA lipid medium (50 ml 1 Mm PA lipid medium is make up of 6 ml 2.13 mg/ml PA, 5 ml 220 mg/ml Bovine serum albumin (BSA) and 39 ml DMEM/F12) for 24 h [17].

### Small interfering RNA and plasmid transfection in vitro

Small interfering RNAs (siRNAs) against LncRNA-gm9795 (GCCTGCATCGGTAATTGAADTDT) were constructed (Genepharma, Suzhou, China) and transfected into AML12 cells by using LipofectaminTM 3000 (Invitrogen, Carlsbad, CA) according to the manufacturer’s instructions. Full-length LncRNA-gm9795 sequence and Flag protein sequence were cloned into the expression vector pCMV (Vigene, Shandong, China), and transfected into AML12 cells according to the manufacturer’s instructions.

### RNA extraction and quantitative real time RT-PCR (qRT-PCR)

Total RNA was extracted from cells or tissues. Quantitative real-time polymerase chain reaction (RT-qPCR) was performed by using the PrimeScript RT Reagent Kit and SYBR Premix Ex Taq (TaKaRa, Dalian, China) following the manufacturer’s instructions. The specific primers used for RT-qPCR are as Table 1. RT-qPCR results were analyzed to obtain CT values of amplified products, and the data was analyzed by the 2-$\Delta {}$ $\Delta {}$CTmethod.

**Table 1:** The specific primers used for RT-qPCR

Gene	Forward	Reverse
gm9795	CCCCTAGAGACTTTATCGACTT	AGTGCAGCCAGGTCTAATTGT
TNF$\alpha {}$	GCCACCACGCTCTTCTGTCTA	GGGTCTGGGCCATAGAACTGAT
IL-1$\beta {}$	GCAACTGTTCCTGAACTCAACT	ATCTTTTGGGGTCCGTCAACT
IL-6	CTCCCAACAGACCTGTCTATAC	CCATTGCACAACTCTTTTCTCA
$\beta {}$-actin	GTGCTATGTTGCTCTAGACTTCG	ATGCCACAGGATTCCATACC
GAPDH	GTTGTCTCCTGCGACTTCA	GCCCCTCCTGTTATTATGG
MALAT1	GCAGCAGTTCGTGGTGAAGATAGG	TCGCCTCCTCCGTGTGGTTG
IRE1$\alpha {}$	GCAGGCTGTGTCTTTTACTATG	TATCAATTCACGAGCAATGACG
CHOP	CTCCAGATTCCAGTCAGAGTTC	ACTCTGTTTCCGTTTCCTAGTT
BIP	ATGATGAAGTTCACTGTGGTGG	CTGATCGTTGGCTATGATCTCC

### Nuclear and cytoplasmic RNA extraction

AML 12 cells were treated according to the manufacturer’s instructions (Thermo Fisher Scientific) to obtain separate nuclear and cytoplasm parts. RNA extraction and quantitative real-time PCR were respectively conducted for nuclear and cytoplasm parts.

### NILE red

After the different treatments, cells were fixed with 4% paraformaldehyde at 26 °C for 15 min, and incubated for 15 min with 1 $\mathrm {\mu }$M Nile red dye working fluid (a hydrophobic dye that accumulates in lipid droplets). Thereafter, DAPI was added to stain the nucleus and the cells were examined by a confocal microscope (Carl Zeiss Company, Germany).

### Western blot

Total proteins were extracted from cells and Westernblot was carried out according to the instructions. Membranes were then incubated with primary antibody [IRE1$\alpha {}$ (1:1000, CST), NF-$\kappa {}$B/p65 (1:1000, CST), p-NF-$\kappa {}$B/p65 (1:1000, CST), JNK (1:1000, Bimake), p-JNK(1:1000, CST), c-Jun(1:1000, CST), p-c-Jun (1:1000, CST), TNF$\alpha {}$ (1:1000 Abcam), $\beta {}$-actin (1:1000, CST), FLAG-tag (1:1000, CST)] according to the manufacturer’s instructions. The ECL chemiluminescence system was used to detect the signals.

### Statistical analysis

All data are presented as means$\pm {}$SD. Statistically significant differences were determined using a Student’s t-test or the Mann-Whitney U test. Statistical analyses were performed using SPSSv.20 (IBM SPSS, Chicago, IL, USA). Values of P < 0.05 were considered significant.

## Results

### Expression profiles of lncRNAs and mRNAs in mice with NAFLD and NASH

**Fig. 1:**
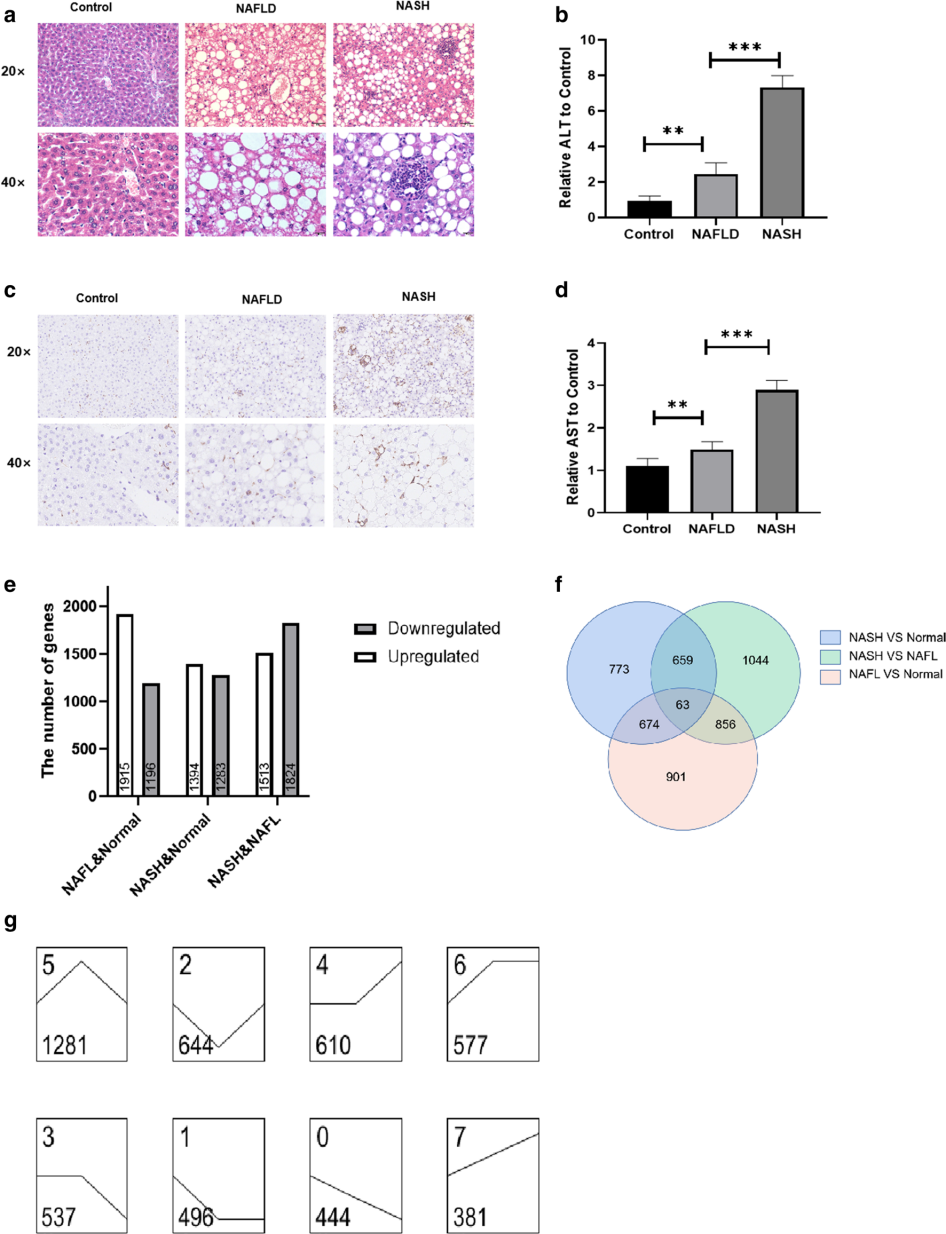
Profiles of lncRNAs in NAFLD, NASH and control mice: **a** HE staining of pathological sections of mice’ liver; ** b** Relative AST level in mice blood (to control); ** c** Immunohistochemical test of F4/80 in pathological sections of mice’ liver; ** d** Relative ALT level in mice blood (to control); ** e** Differences in lncRNAs between NAFLD, NASH and Normal mice; **f** The venn analysis of differential lncRNAs of NAFLD, NASH and control mice; (**g**). The series cluster analysis of differential lncRNAs of NAFLD, NASH and control mice: The left dot represents the normal group, the middle dot represents the NAFLD group, and the right dot represents the NASH group. The top left number is the number of trends, and the bottom left is the number of different genes; (**P < 0.01,***P < 0.001)

To determine the expression profiles of lncRNAs and mRNAs in mice with NAFLD and NASH, the Mouse LncRNA Microarray V2.0 (Arraystar, Rockville, MD, United States) were used. Pathological sections showed that fat accumulation was significantly increased in DB/DB mice fed with normal diet(NAFLD model) compared with the control group, while DB/DB mice fed with MCD diet(NASH model) showed significant inflammatory cell infiltration, indicating the success of the model building (Fig. 1a). F4/80(EMR1) is a heavily glycosylated G-protein-coupled receptor and is a well-established marker for mouse macrophages [18]. IHC with F4/80 showed significant inflammation in NASH mice (Fig. 1c). The blood tests of mice showed that ALT in NASH group were 2.98 times higher than control group and AST were 1.94 times higher than control group (Fig. 1b, d). The results of Microarray showed that a total of 3111 lncRNAs (1915 upregulated and 1196 downregulated) were differentially expressed between the NAFLD and control groups. There were 1394 upregulated lncRNAs and 1283 downregulated lncRNAs were found between the NASH and control groups, while 1513 upregulated and 1824 downregulated lncRNA were found between the NASH and NAFLD groups (Fig. 1e). Venn analysis was performed on the differentially expressed lncRNAs in the three groups to find the common and unique lncRNAs in the three groups. Results shows that there are 63 lncRNAs changed in all the three groups (Fig. 1f). And The results of Series Cluster found that there are 381 lncRNAs were found to be not only highly expressed in NAFLD, but also is going to go even higher in NASH (Fig.1g), which caught our attention. Among these genes, we focus on a novel lncRNA-gm9795 and has carried on the thorough research to it.

### LncRNA-gm9795 has no coding capacity and is located in the nucleus

**Fig. 2:**
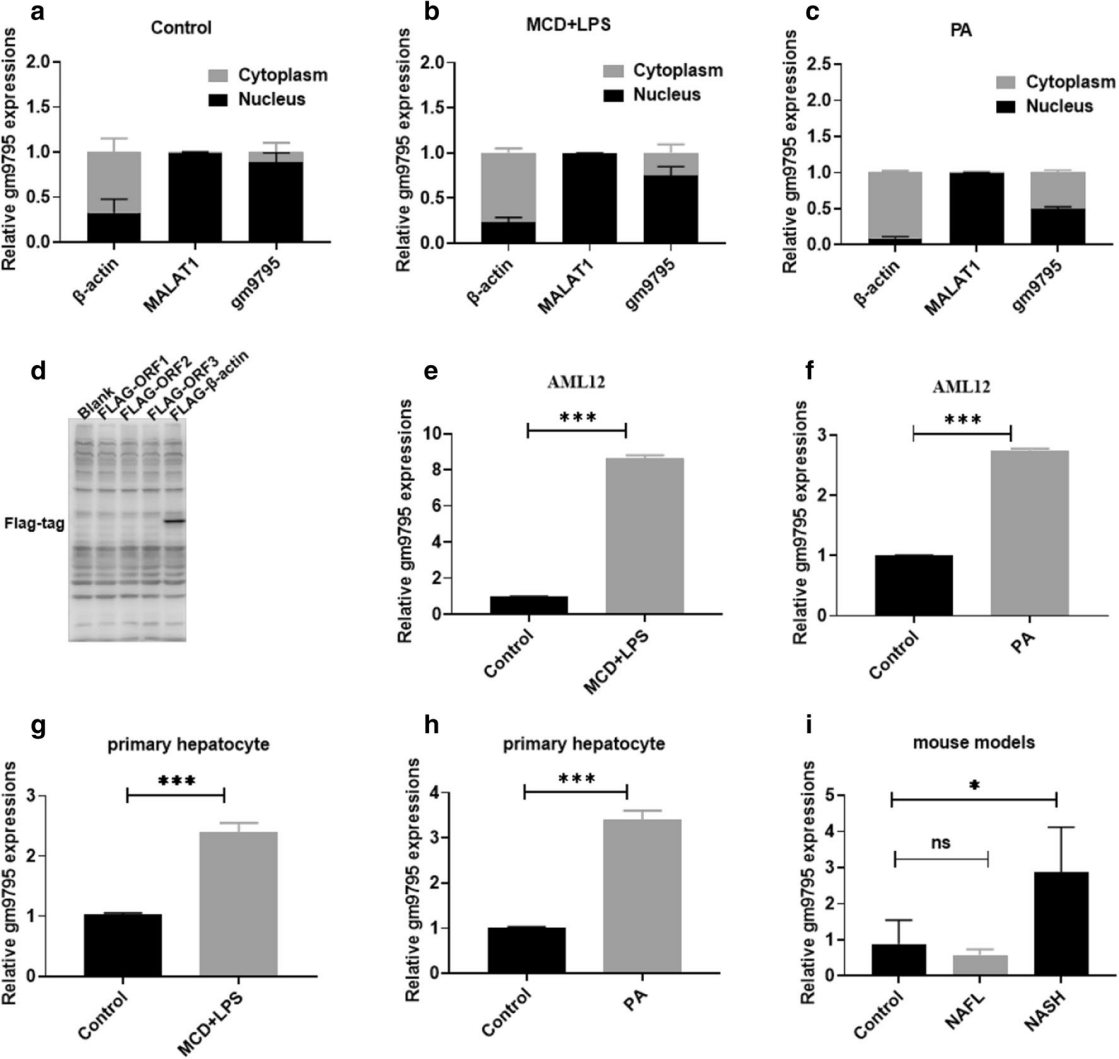
LncRNA-gm9795 is highly expressed in liver tissues of NASH mice and cell models: **a** LncRNA-gm9795 mainly in the nucleus in control group; **b**. LncRNA-gm9795 mainly in the nucleus in MCD+LPS group; **c** LncRNA-gm9795 mainly in the nucleus in PA group; **d** Three Open Read Frames of LncRNA-gm9795 predicted in the database showed no coding capacity; **e** LncRNA-gm9795 is highly expressed in NASH cell models built by MCD+LPS in AML12; **f** LncRNA-gm9795 is highly expressed in NASH cell models built by PA in AML12; **g** LncRNA-gm9795 is highly expressed in NASH cell models built by MCD+LPS in mouse primary hepatocytes; **h** LncRNA-gm9795 is highly expressed in NASH cell models built by PA in mouse primary hepatocytes; **i** LncRNA-gm9795 is highly expressed in NASH mouse models; (NS: Nonsense, *P < 0.05, **P < 0.01,***P < 0.001)

In order to further understand LncRNA-gm9795, we first studied its basic characteristics. With the method of separating nucleoplasm RNA, its expression in the nucleus and cytoplasm was detected. LncRNA - MALAT1 was recruited as the nucleus marker gene, while beta actin was used as the cytoplasmic marker gene. The results showed that LncRNA-gm9795 was mainly distributed in the nucleus all in the control group (Fig. 2a), MCD+LPS group (Fig. 2b) and PA group (Fig. 2c). And it tended to emerge from the nucleus to cytoplasm after modeling. Then we connected the full length of LncRNA-gm9795 with the FLAG sequence to construct plasmids. The expression of FLAG tag was detected after the transfection into normal mouse liver cells, and Actin connected with the FLAG sequence was used as the positive control. The results showed that the expression of FLAG tag could be detected by the transfection of Actin-Flag, while the expression of FLAG tag was not detected by the transfection of LncRNA-gm9795- FLAG (Fig. 2d).

### LncRNA-gm9795 is highly expressed in liver tissues of NASH mouse and cell models

MCD medium combined with 1 $\mu {}$g/ml LPS and 1 mM PA were used to stimulate AML12 cells and primary mouse liver cells to construct NASH in vitro model, and the mRNA expression level of LncRNA-gm9795 in vitro model was verified by RT-qPCR. Similarly, LncRNA-gm9795 increased about 5 times in the MCD+LPS AML12 cell model (Fig. 2e) and 2 times in the PA AML12 cell model (Fig. 2f), and the results were also validated in the NASH model of primary hepatocytes (Fig. 2g, h) (P < 0.05). Meanwhile, We used male C57 BLKS/J db/db mice with normal and MCD diet to construct mouse models of NAFL and NASH respectively, with C57 BLKS/J mice feeding normal diet as the control group. RNA was extracted from the liver tissues of mice in each group and the expression of LncRNA-gm9795 was detected by qRT-qPCR. The results showed that the mRNA level of LncRNA-gm9795 in NASH liver tissues was about 6 times higher than that in normal liver tissues (Fig. 2i).

### LncRNA-gm9795 does not affect fat accumulation in NASH

**Fig. 3:**
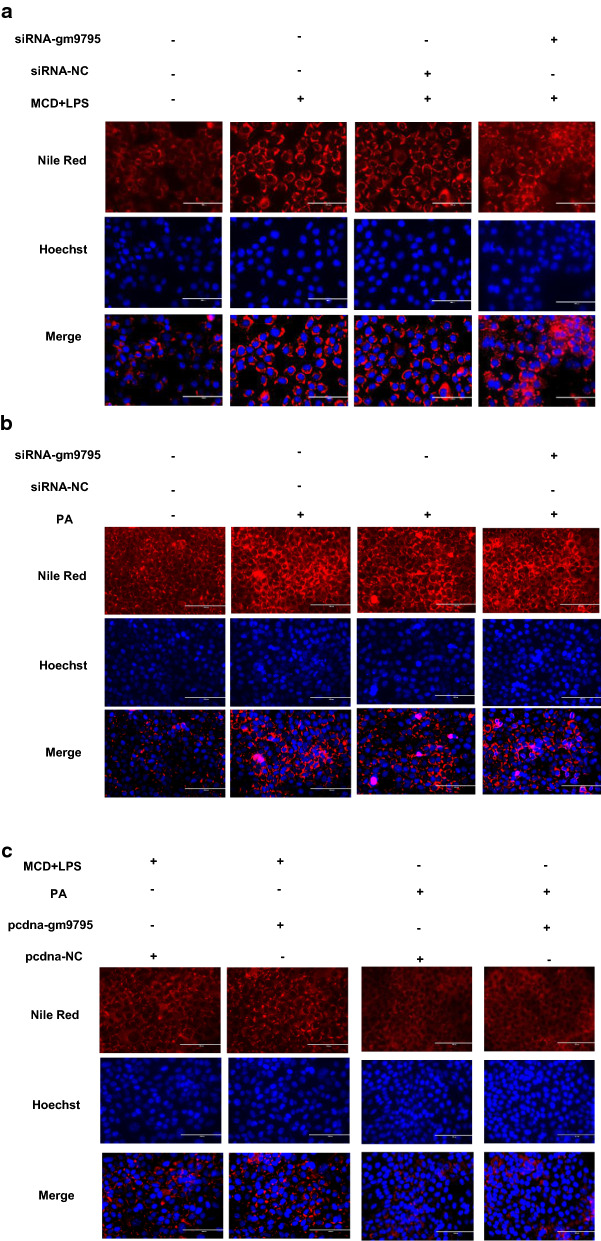
lncRNA-gm9795 did not affect lipid accumulation in NASH: ** a** Fluorescence imaging shows there was no significant change in lipid deposition after interference with lncRNA-gm9795 in MCD+LPS model; **b** Fluorescence imaging shows there was no significant change in lipid deposition after interference with lncRNA-gm9795 in PA model; ** c** Fluorescence imaging shows there was no significant change in lipid deposition after overexpressing lncRNA-gm9795 in NASH model

In order to determine whether LncRNA-gm9795 affects lipid accumulation during the pathogenesis of NASH, MCD+LPS and PA were respectively constructed NASH model after interfering LncRNA-gm9795 in normal AML12, and the lipid deposition was observed in cells by fluorescent imaging using Nile red staining. Fluorescence imaging showed that the red fluorescence increased after the MCD+LPS and PA model was constructed, indicating that the lipid deposition increased. However, the interference with lncRNA-gm9795 did not decrease the red fluorescence (Fig. 3a, b). Similar phenomena can be observed with over-expression of LncRNA-gm9795 (Fig. 3c).

### LncRNA-gm9795 significantly increases the expression of inflammation factors in NASH through NF-kB/JNK pathway

**Fig. 4:**
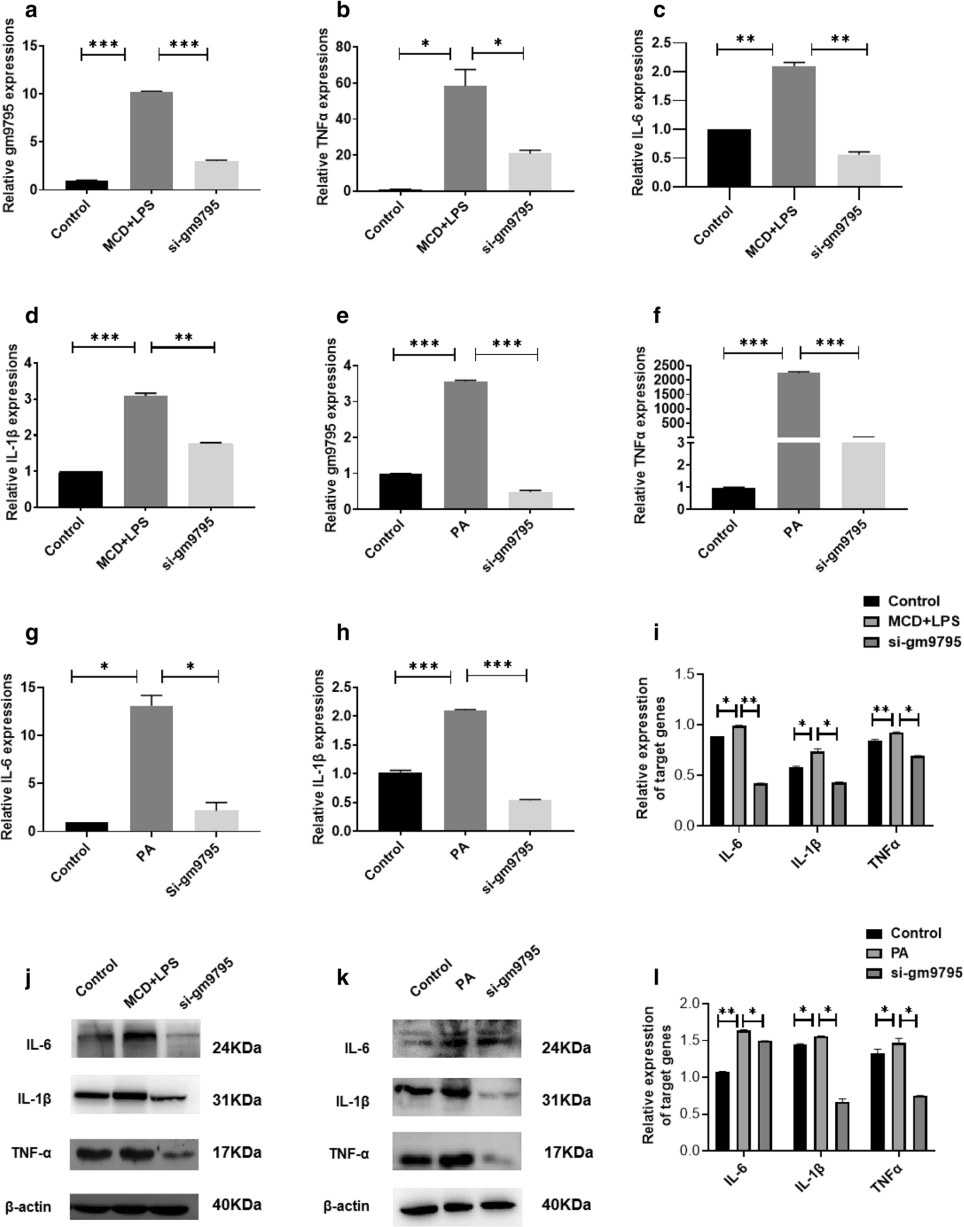
Knocking down LncRNA-gm9795 significantly decreased the expression of inflammation factors in NASH: ** a** Interference efficiency of siRNA-gm9795 in MCD+LPS model; ** b** In MCD+LPS model, the mRNA expression of TNF$\alpha {}$ was decreased after knocking down gm9795; **c** In MCD+LPS model, the mRNA expression of IL-6 was decreased after knocking down gm9795; **d** In MCD+LPS model, the mRNA expression of IL-1$\beta {}$ was decreased after knocking down gm9795; **e** Interference efficiency of siRNA-gm9795 in PA model; f. In PA model, the mRNA expression of TNF$\alpha {}$ was decreased after knocking down gm9795;g. In PA model, the mRNA expression of IL-6 was decreased after knocking down gm9795; **h** In PA model, the mRNA expression of IL-1$\beta {}$ was decreased after knocking down gm9795; ** i** Relative gray analysis of protein expression of inflammation factors with gm9795 interference in MCD+LPS model; ** j** The protein expression of inflammation factors was decreased after knocking down gm9795 in MCD+LPS model; ** k** The protein expression of inflammation factors was decreased after knocking down gm9795 in PA model; ** l** Relative gray analysis of protein expression of inflammation factors with gm9795 interference in PA model; (*P < 0.05, **P < 0.01,***P < 0.001)

The expression of LncRNA-gm9795 in normal mouse liver cells-AML12 was interfered with siRNA in two NASH models. The interference efficiency and the mRNA expression of inflammatory factors TNF$\alpha {}$, IL-6 and IL-1$\beta {}$ were detected by qPCR. Western blot was used to detect the protein expression of the above factors. Results showed that LncRNA-gm9795 was downregulated effectively (Fig. 4a, e), and the mRNA expression of TNF$\alpha {}$,IL-6 and IL-1$\beta {}$ was reduced when silencing the expression of LncRNA-gm9795 (Fig. 4b–d, f–h). Western Blot results showed that the expression of TNF$\alpha {}$,IL-6 and IL-1$\beta {}$ in both the two NASH models was increased, and their expression was significantly decreased after interfering with LncRNA-gm9795 (Fig. 4i–l).

**Fig. 5:**
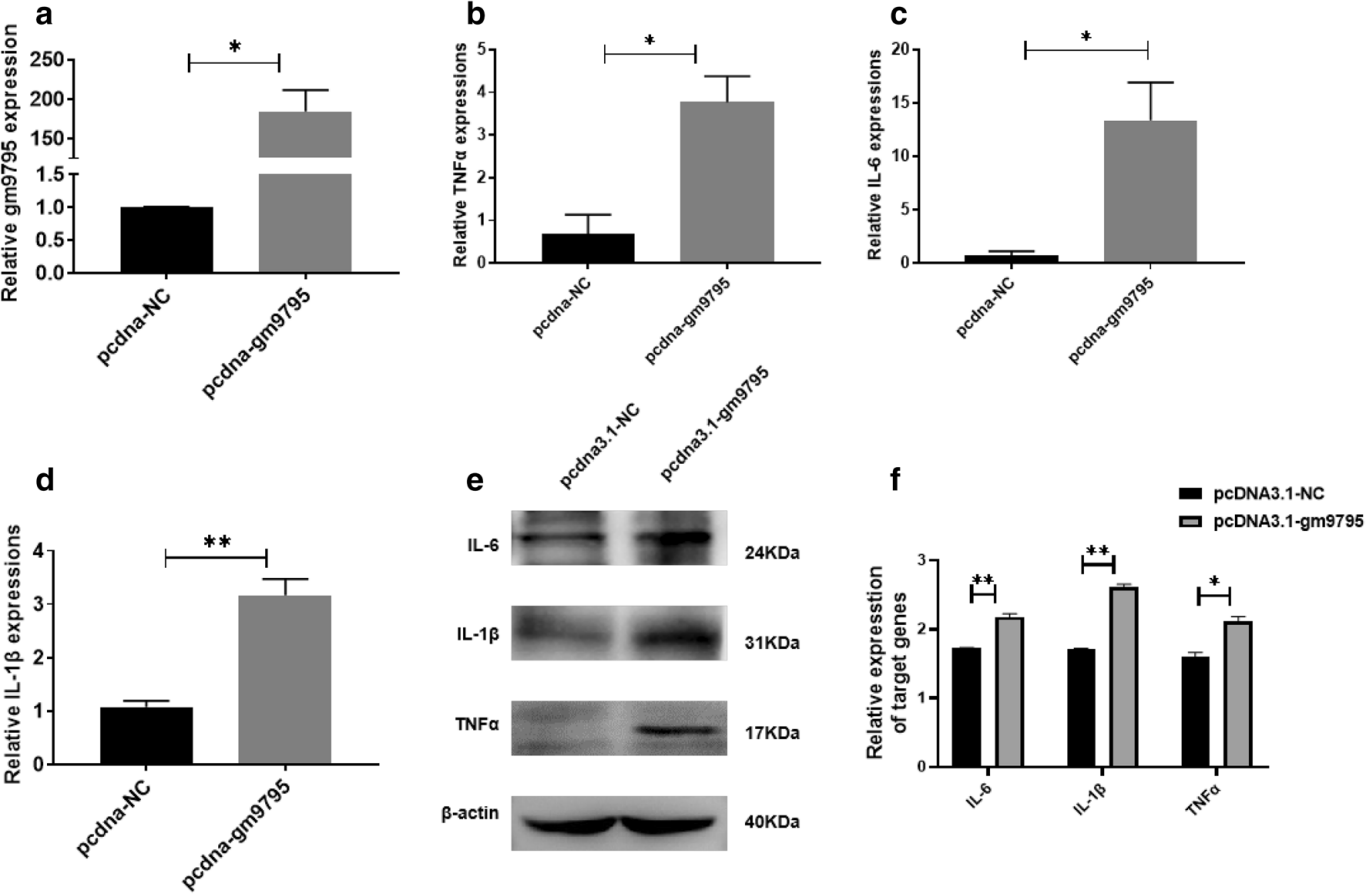
LncRNA-gm9795 significantly increased the expression of inflammatory factors in NASH: ** a** The efficiency of gm9795 overexpression plasmid; ** b** The mRNA expression of TNF$\alpha {}$ increased after the overexpression of gm9795; ** c** The mRNA expression of IL-6 increased after the overexpression of gm9795; ** d**The mRNA expression of IL-1$\beta {}$ increased after the overexpression of gm9795; ** e** The protein expression of inflammatory factors increased after the overexpression of gm9795; ** f** Relative gray analysis of protein expression of inflammation factors with gm9795 overexpression; (*P < 0.05, **P < 0.01,***P < 0.001)

To further understand its biological function. the LncRNA-gm9795 overexpression plasmid was transfected into AML12 cells. The overexpress efficiency and the expression of inflammatory factors were detected by qPCR, and the protein levels were detected by Western Blot. After the transfection with LncRNA-gm9795 overexpression plasmid, the mRNA level of gm9795 in AML12 cells was increased by more than 150 times (Fig. 5a), which proved that the transfected plasmid was successful. TNF$\alpha {}$, IL-6 and IL-1$\beta {}$mRNA level in the overexpression group was about 3 times higher than that in the normal control group by qPCR (Fig. 5b–d). Western Blot results showed that the expression of inflammation factors was significantly up-regulated in the overexpression group (Fig. 5e, f).

**Fig. 6:**
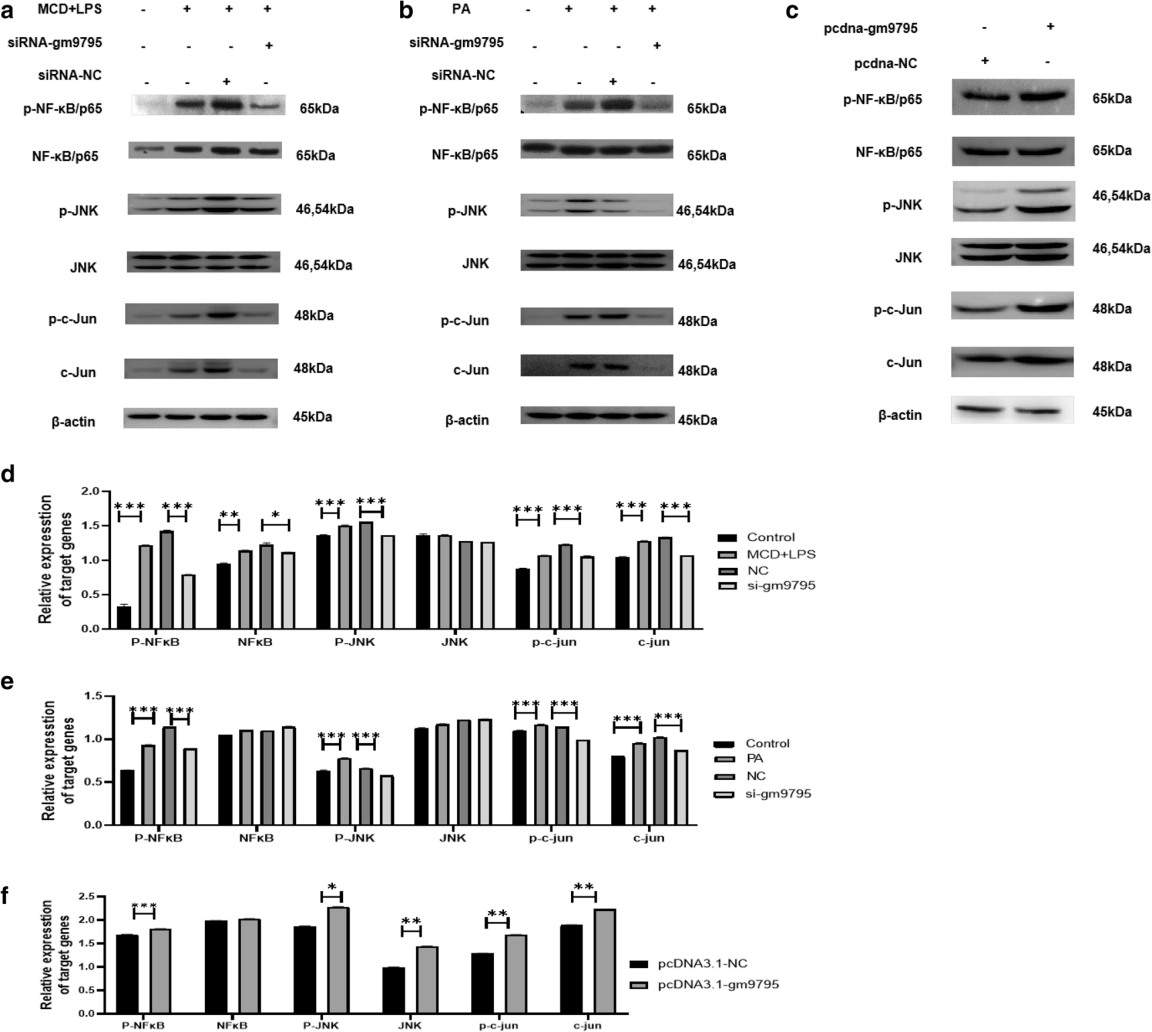
LncRNA-gm9795 significantly increased the expression of inflammation factors in NASH through NF-k*κ*B/JNK pathway: ** a** The expression of NF-$\kappa {}$B/JNK signaling proteins was decreased after gm9795 knockout in MCD+LPS model; ** b** The expression of NF-$\kappa {}$B/JNK signaling proteins was decreased after gm9795 knockout in PA model; ** c** The expression of NF-$\kappa {}$B/JNK signaling proteins was increased after the overexpression of gm9795; ** d** Relative gray analysis of protein expression of NF-$\kappa {}$B/JNK with gm9795 interference in MCD+LPS model; ** e** Relative gray analysis of protein expression of NF-$\kappa {}$B/JNK with gm9795 interference in PA model; ** f** Relative gray analysis of protein expression of NF-$\kappa {}$B/JNK with gm9795 overexpression; (*P < 0.05, **P < 0.01,***P < 0.001)

Inflammatory response is an important link in NASH. After changing the expression of LncRNA-gm9795, the protein levels of classic inflammatory pathway proteins NF-k$\kappa {}$B/p65, JNK and c-Jun were detected by Western Blot. Results showed that the expression of NF-k*κ*B/p65, p-NF-k*κ*B/p65, JNK, p-JNK, c-Jun and p-c-Jun in both the two NASH model were increased, and the expression levels of the above proteins were significantly decreased after interfering with LncRNA-gm9795 (Fig. 6a, b). LncRNA-gm9795 overexpression plasmid was transfected into AML12 cells and western Blot results showed that the expression of p-NF-k*κ*B/p65, p-JNK, c-Jun and p-c-Jun was significantly up-regulated in the overexpression group (Fig. 6c). Thus, it is clear that LncRNA gm9795 up-regulated inflammation factors expression by activating NF-k*κ*B/JNK signaling pathway.

### LncRNA-gm9795 activates NF-k*κ*B/JNK pathway by activating ERS

**Fig. 7:**
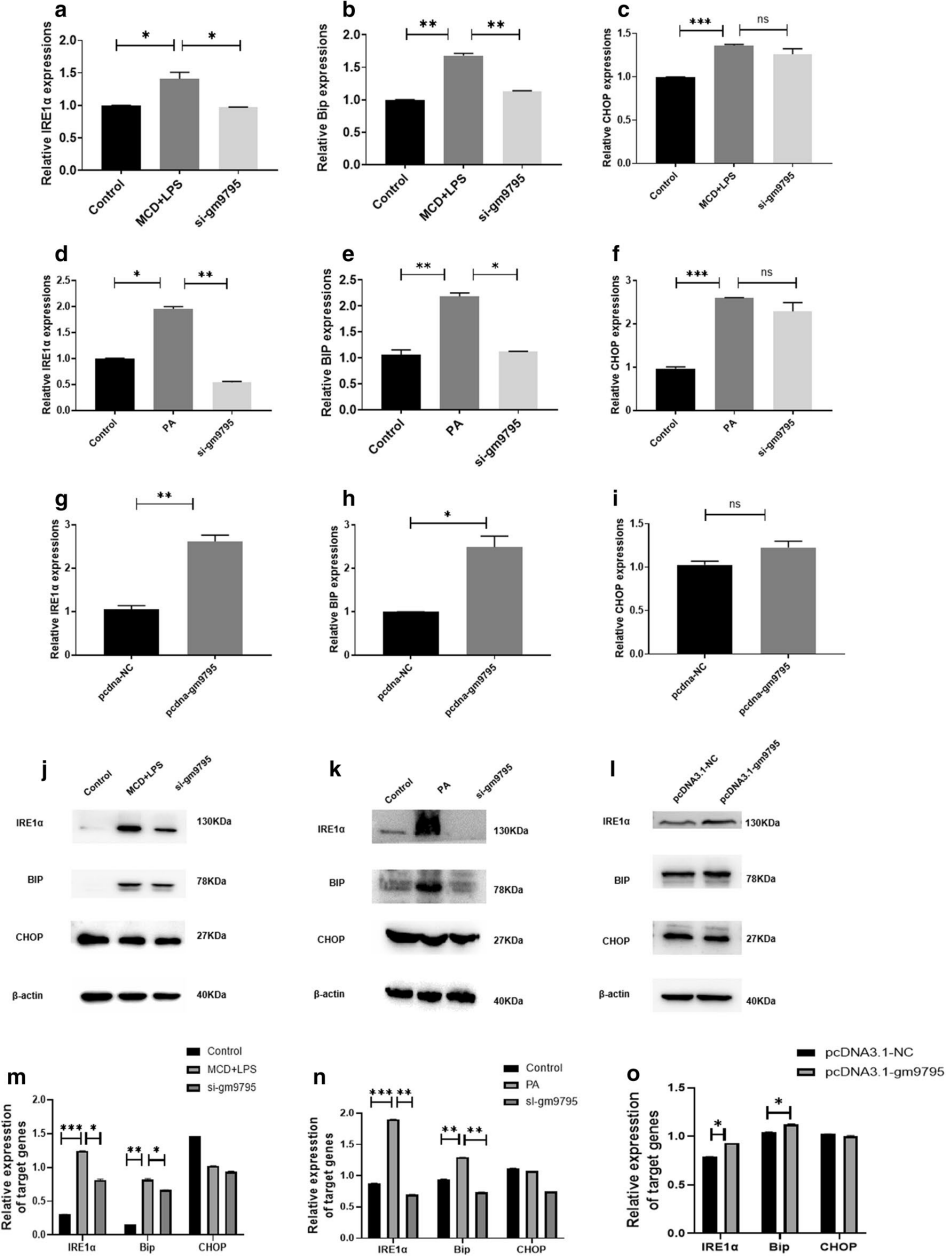
LncRNA-gm9795 activates ERS: ** a** The mRNA expression of IRE1$\alpha {}$ was decreased after gm9795 knockout in MCD+LPS model; ** b** The mRNA expression of BIP was decreased after gm9795 knockout in MCD+LPS model; ** c** The mRNA expression of CHOP had no significant change after gm9795 knockout in MCD+LPS model; ** d** The mRNA expression of IRE1$\alpha {}$ was decreased after gm9795 knockout in PA model; ** e** The mRNA expression of BIP was decreased after gm9795 knockout in PA model; ** f** The mRNA expression of CHOP had no significant change after gm9795 knockout in PA model; ** g** The mRNA expression of IRE1$\alpha {}$ were increased with gm9795 overexpression; ** h** The mRNA expression of BIP were increased with gm9795 overexpression; ** i** The mRNA expression of CHOP had no significant change with gm9795 overexpression; ** j** The protein expression of ERS was decreased after gm9795 knockout in MCD+LPS model; ** k** The protein expression of ERS was decreased after gm9795 knockout in PA models; ** l** The protein expression of ERS was were increased with gm9795 overexpression; ** o** Relative gray analysis of protein expression of ERS with gm9795 interference in MCD+LPS model; ** e** Relative gray analysis of protein expression of ERS with gm9795 interference in PA model; ** f** Relative gray analysis of protein expression of ERS with gm9795 overexpression; (*P < 0.05, **P < 0.01,***P < 0.001)

ERS, as the current studies say, is an important part of the evolution of NASH. Thus, the expression of ERS-related molecules in the NASH models with LncRNA-gm9795 overexpression and knockout was detected. Results showed that after LncRNA-gm9795 was knocked out, the expression of IRE1$\alpha {}$ and Bip was down-regulated, while no significant change was observed in CHOP (Fig. 7a–f, j–k, m–n). qPCR and Western Blot results also showed that the expression of IRE1$\alpha {}$ and Bip was significantly up-regulated in the overexpression group (Fig. 7g–i, l, o).

**Fig. 8:**
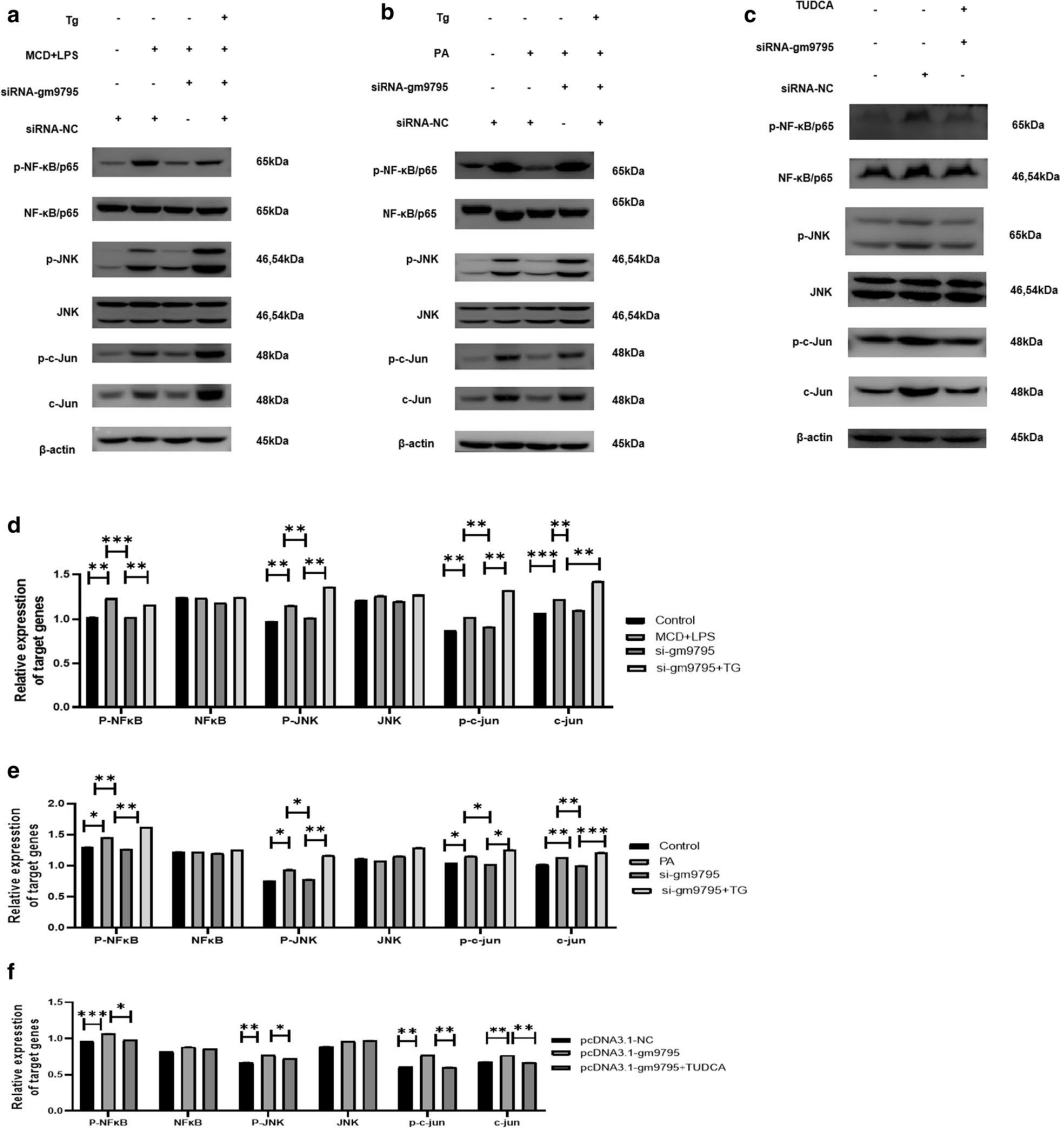
LncRNA-gm9795 activated NF-k*κ*B/JNK pathway through ERS: ** a** The NF-k*κ*B/JNK pathway was activated by introducing endoplasmic reticulum stress agonists after gm9795 was knocked out in MCD+LPS model; ** b** The NF-k*κ*B/JNK pathway was activated by introducing endoplasmic reticulum stress agonists after gm9795 was knocked out in PA model; ** c** The NF-k*κ*B/JNK pathway was inhibited by introducing endoplasmic reticulum stress inhibitor after gm9795 was overexpressed; ** d** Relative gray analysis of protein expression of NF$\kappa {}$B/JNK with gm9795 interference and TG in MCD+LPS model; ** e** Relative gray analysis of protein expression of NF$\kappa {}$B/JNK with gm9795 interference and TG in PA model; **f** Relative gray analysis of protein expression of NF$\kappa {}$B/JNK with gm9795 overexpression and TUDCA; (*P < 0.05, **P < 0.01,***P < 0.001)

To determine whether LncRNA-gm9795 up-regulates the inflammatory factors by activating NF-kB/JNK signaling pathway through ERS, after interfering with LncRNA-gm9795, MCD+LPS and PA were used to establish NASH models, and endoplasmic reticulum stress agonist Thapsigargin (Tg) (800 nM, as the dosage recommended in the instructions) was added to stimulate the models for 24h. Western Blot showed that the addition of Tg could significantly reverse the effect of interfering LncRNA-gm9795 on the proteins of the IRE1$\alpha {}$/NF-kB p65/JNK pathway (Fig. 8a, b). At the same time, we transfected LncRNA-gm9795 overexpression plasmid into AML12 cells and added the endoplasmic reticulum stress inhibitor TUDCA (0.2mM, as the dosage recommended in the instructions) to stimulate them for 24h after transfection. Western Blot showed that TUDCA could antagonize the up-regulation of IRE1$\alpha {}$/NF-k*κ*B/JNK pathway caused by the overexpression of LncRNA-gm9795 (Fig. 8c).

## Discussion

In recent years, LncRNA has attracted more and more attentions. Studies have shown that lncRNA plays a key role in regulating gene expression such as chromatin modification, transcription and post-transcriptional processing, and its abnormal expression is related to many physiological and pathological processes, such as embryonic development, cell proliferation and differentiation, steatosis, oxidative stress, endoplasmic reticulum stress, etc [19, 20]. One of the first investigations of lncRNAs in NAFLD fibrosis demonstrated that MEG3 expression was decreased in livers from CCl4-treated mice compared to oil-fed control mice, and Meg3 expression diminished concordantly with the progression of fibrosis [21]. In an independent study, Aptr expression was found to be more than twofold higher in fibrotic livers of two animal models for liver fibrosis (CCl4 and bile duct ligation (BDL) mice) and in humans with liver fibrosis of undisclosed etiology [22]. And hepatic Malat1 expression was upregulated in hepatic stellate cells and hepatocytes respectively isolated from CCl4-treated animals when compared to control mice [23]. There’s no doubt that lncRNAs play an important role in NASH. Our study found a new LncRNA-gm9795, which is specifically high expressed in NASH. It is consistent with the results of our previous gene chip. We found through experiments that the lncRNA had no coding ability and was mainly distributed in the nucleus. It lays a solid foundation for us to further study its functions.

The specific pathogenesis of NAFLD is complex, and no definite conclusion has been reached by domestic and foreign scholars. Both the two hypothesis of NAFLD emphasized inflammation in NASH [24]. The accumulation of a variety of inflammatory cytokines (such as TNF$\alpha {}$, IL-1, IL-6, etc.) causes liver inflammation and liver cell damage, and then progresses to liver fibrosis [25]. The present study found that LncRNA-gm9795 up-regulates the expression of Inflammatory cytokines in NASH, in both the NASH in vitro models, indicating that LncRNA-gm9795 promotes inflammatory reaction in NASH.

In recent years, the “multiple hit” theory highlights the key role of ER stress in NAFLD, especially in NASH [26]. Endoplasmic reticulum (ER) is the site of protein synthesis, folding, transit and modification. ER is extremely sensitive to various stimuli, including hypoxia, hyperglycemia, chemical poisons and other factors, which may lead to the accumulation of misfolded and unfolded proteins in the lumen of ER and causing ERS. The first reaction occurs in ERS is that the up-regulation of molecular chaperone glucose regulating protein 78 (GRP78) improves protein folding or corrects misfolding [27]. However, the binding of GRP78 and unfolded proteins results in its dissociation with the stress sensor protein kinase RNA-like endoplasmic reticulum kinase (PERK) and inositol requiring enzyme 1$\alpha {}$ (IRE1$\alpha {}$), which causes the activation of these proteins and triggers ERS. After the dissociation of PERK with GRP78, PERK is activated by dimerization and autophosphorylation, which phosphorylates the downstream factor eukaryotic translation initiation factor 2$\alpha {}$ (EIF2$\alpha {}$), thereby forming P-EIF2$\alpha {}$. P-EIF2$\alpha {}$ regulates the activation of NF-$\kappa {}$B via reducing the synthesis of the inhibitor of NF-$\kappa {}$B (I$\kappa {}$B) [3, 28, 29]. Therefore, ERS-related inflammation plays an important role in the development of insulin resistance, liver injury, and hepatic fibrosis in NASH. Our study found that LncRNA-gm9795 in NASH in vitro model significantly activates the expression of IRE1$\alpha {}$ and BIP, which are key molecules of endoplasmic reticulum stress. However, CHOP, an important pro-apoptotic molecule in ERS, did not change significantly. Both flow cytometry and western blot showed that it had no significant effect on apoptosis after gm9795 interference. Furthermore, the NF-k*κ*B/JNK pathway related proteins were also found activated. Gm9795 does not affect apoptosis in NASH in our data, which suggested that phosphorylated JNK/ c-Jun promotes the development of NASH mainly through activation of inflammation. And we found that it can be restored by an agonist or inhibitor of ERS. It means LncRNA-gm9795 might activate NF-k*κ*B/JNK pathway by activating ERS, finally resulting in inflammation. It wound be a new finding in the development of NASH, which might provide a new therapeutic target.

**Fig. 9:**
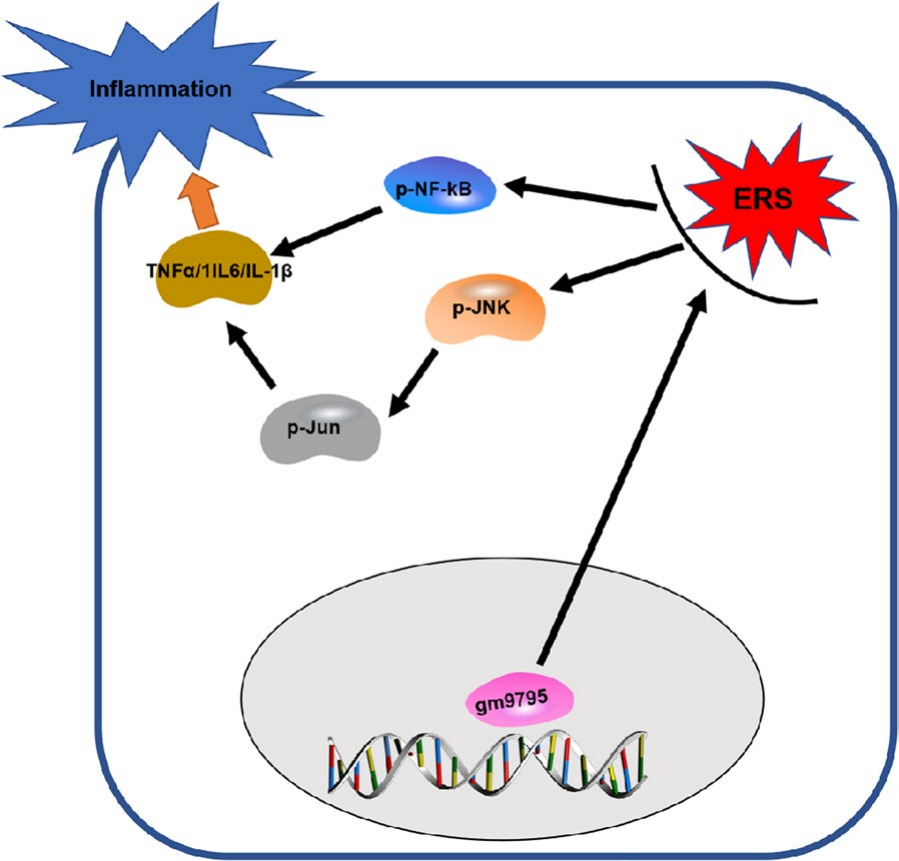
Diagram of LncRNA-Ggm9795 promoting inflammation in NASH:LncRNA-gm9795 activated NF-k*κ*B/JNK pathway through ERS

## Conclusion

The present study found a novel LncRNA-gm9795,which was significantly up-regulated in vivo and in vitro models. The abnormal high expression of LncRNA-gm9795 could enhance ERS, activate the signaling pathway of NF-$\kappa {}$B /JNK, and subsequently affect the expression of inflammatory factors (Fig 9). These findings help us better understand the various mechanisms of LncRNA-gm9795, which may become a potential prognostic indicator and therapeutic target for NASH.

## Data Availability

The datasets used and/or analysed during the current study are available from the corresponding author on reasonable request.

## Abbreviations

ITS: Insulin-transferrin-selenium
DM: Dexamethasone
MCD: Methionine-choline-deficient medium
LPS: Lipopolysaccharide
PA: Palmitic acid
ERS: Endoplasmic reticulum stress
TG: Thapsigargin
TUDCA: Tauroursodeoxycholic acid
